# Improved sub-genomic RNA prediction with the ARTIC protocol

**DOI:** 10.1093/nar/gkae687

**Published:** 2024-08-16

**Authors:** Thomas Baudeau, Kristoffer Sahlin

**Affiliations:** Univ. Lille, CNRS, Centrale Lille, UMR 9189 CRIStAL, F-59000 Lille, France; Department of Mathematics, Science for Life Laboratory, Stockholm University, 106 91 Stockholm, Sweden

## Abstract

Viral subgenomic RNA (sgRNA) plays a major role in SARS-COV2’s replication, pathogenicity, and evolution. Recent sequencing protocols, such as the ARTIC protocol, have been established. However, due to the viral-specific biological processes, analyzing sgRNA through viral-specific read sequencing data is a computational challenge. Current methods rely on computational tools designed for eukaryote genomes, resulting in a gap in the tools designed specifically for sgRNA detection. To address this, we make two contributions. Firstly, we present sgENERATE, an evaluation pipeline to study the accuracy and efficacy of sgRNA detection tools using the popular ARTIC sequencing protocol. Using sgENERATE, we evaluate periscope, a recently introduced tool that detects sgRNA from ARTIC sequencing data. We find that periscope has biased predictions and high computational costs. Secondly, using the information produced from sgENERATE, we redesign the algorithm in periscope to use multiple references from canonical sgRNAs to mitigate alignment issues and improve sgRNA and non-canonical sgRNA detection. We evaluate periscope and our algorithm, periscope_multi, on simulated and biological sequencing datasets and demonstrate periscope_multi’s enhanced sgRNA detection accuracy. Our contribution advances tools for studying viral sgRNA, paving the way for more accurate and efficient analyses in the context of viral RNA discovery.

## Graphical Abstract

**Graphical Abstract:**
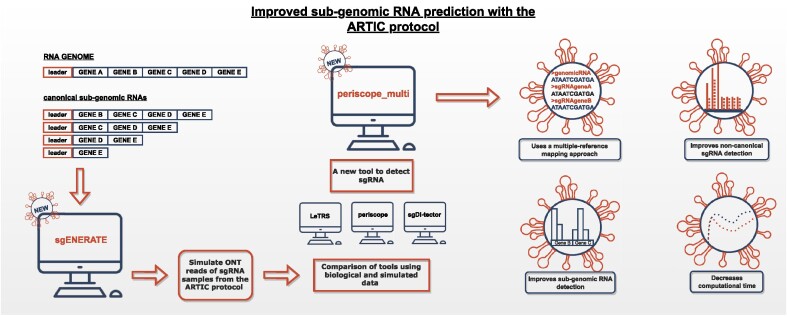

## Introduction

**Figure 1.**
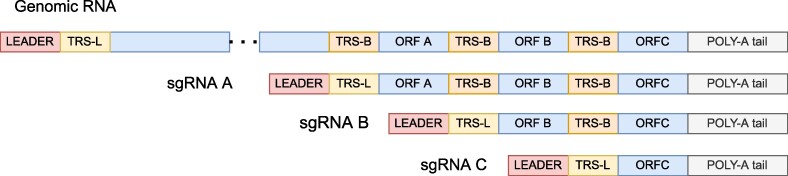
Schematic representation of SARS-COV2 genomic RNA and sgRNA. Top sequence shows the full genomic RNA. The bottom three sequences are three examples of the generated sgRNA for the A, B and C genes.

Among the biological mechanisms specific to viruses is subgenomic RNA (sgRNA) production. Subgenomic RNA is produced by some of the RNA-positive viruses in the Baltimore classification. Although not all RNA-positive viruses use this mechanism, there are a number of species, including one of interest which encompasses viruses of the *Coronaviridae* family, counting SARS-COV-2 (1), responsible for the COVID-19 pandemic. Previous studies (2,3) have shown the major role of sgRNA in the cycle of replication of the virus and claim that sgRNA also influences the pathogenicity and may have an influence on the evolution of the viruses. The process of generating viral sgRNAs, such as in SARS-COV2, involves a sequence called the ‘leader’ followed by a transcription regulatory sequence (TRS), which can be found at the start of the genomic RNA (Figure 1). Following this sequence are the open reading frames (ORFs) of genes. To produce sgRNAs, biological mechanisms will cut inside the sequence to connect the leader sequence to one of the ORFs (Figure 1). The sgRNAs are categorized as canonical when they are shared among the viral population (e.g. as for genes S, E, M, N, ORF3a, ORF6, ORF7a, ORF8, ORF10 for SARS-COV2). There are also non-canonical (4,5) sgRNAs, that are sporadically produced, with non-canonical sgRNAs being a minority (2). The non-canonical sgRNAs provide diversity in viral RNA regulation and differ from canonical sgRNAs in junction sites. They can also differ in the biological mechanism to produce them. From a bioinformatic perspective, the sgRNA process can be thought of as alternative splicing in that it merges together different segments from the genome, albeit through other underlying biological mechanisms. Various methods are employed for sequencing viral genomes to avoid the limitations of shotgun sequencing, which can generate reads from multiple sources, including the host, microbiota and the virus. One alternative is culturing the virus in a controlled laboratory environment, which can reduce biases but may introduce artificial mutations. An effective method is virus PCR amplification (6), offering safer and simplified analyses by amplifying only the viral genome, overcoming shotgun sequencing’s limitations. One such RT-PCR amplification is the ARTIC protocol (7,8), which has proven effective in monitoring viral propagation during epidemics (7,9–12). Both Next Generation Sequencing (NGS), such as Illumina, and Third Generation Sequencing (TGS), such as Oxford Nanopore Technologies (ONT), can be used to sequence the ARTIC protocol. While Illumina sequencing still provides better detection for variant calling thanks to superior read quality and coverage, ONT is rapidly evolving in throughput and read quality. In addition, ONT light-weight devices and long reads enable portability and the ability to sequence the amplicons in full, which makes it particularly interesting for viral monitoring. The shorter sequencing time also allows about five times more samples to be sequenced compared to Illumina sequencing, which is an advantage for tracking epidemics, as discussed in (13).

However, there are computational challenges in analyzing ARTIC ONT cDNA data. The reads have a high error rate due to both amplification-based errors and sequencing errors. Furthermore, the amplicon-sequenced reads are relatively short for long-read technologies, making them, together with errors, challenging to map. So far, there have been two primary approaches to analyzing viral sgRNA data. The first employs read mappers in splice alignment mode, such as STAR(14) for NGS data and minimap2 (15) for TGS data (2,4,16,17). However, these tools are optimized for eukaryotes and may produce spurious alignments by introducing artificial gaps in reads. Additionally, spliced alignment is computationally intensive (15). There is also a tool, sgDI-tector (18), that performs its own customized spliced mapping approach, but it is designed only for NGS data. The second approach involves a two-step procedure, as demonstrated by periscope (19). This method first filters out contamination by mapping reads to a viral reference genome, and the second step scans mapped reads for the leader sequence through local alignment. While periscope is a welcomed tool for the detection of sgRNA and overcomes eukaryote-designed spliced-alignment-related issues, it has its limitations, including alignment bias from aligning to a linear reference genome (20,21) and unnecessary computational time requirements.

Overall, the ARTIC protocol with ONT cDNA sequencing offers excellent promise for viral monitoring during an epidemic, but computational challenges arise due to errors in amplicon-derived noisy reads. The computational challenges of analyzing ARTIC data prompted us to assess the accuracy of detecting canonical and non-canonical sgRNAs for tools using ONT-based ARTIC protocol sequencing. We designed an evaluation pipeline, sgENERATE, to study the accuracy and efficiency of sgRNA detection pipelines based on the ARTIC protocol and detected that the computational approach, periscope, has limitations. While periscope is the only dedicated tool that currently can analyze sgRNA reads from ARTIC protocol, sgENERATE is modular and can integrate additional ONT sequencing ARTIC-based tools as they become available. Such a pipeline can help to evaluate future bioinformatic pipelines for ARTIC data and, through feedback on simulated data, speed up the development of computational pipelines for analyzing such sequencing data. We demonstrate this by redesigning periscope to overcome the limitations we observed based on the observations from our evaluation pipeline. We call our redesigned approach periscope_multi. Periscope_multi has the same interface as periscope, but uses multiple references from all the canonical sgRNA to overcome the splice alignment issues. We evaluated periscope and periscope_multi using simulated and biological sequencing datasets and show that periscope_multi improves both sgRNA and non-canonical sgRNA detection while being over three times faster. Finally, we discuss current limitations and future directions for computational improvement of viral RNA discovery.

## Materials and methods

### The periscope pipeline

Periscope (19) is a Python pipeline developed to find sub-genomic RNA in both ONT and Illumina reads using the ARTIC (7) protocol. The ARTIC protocol is an amplicon-based sequencing protocol designed for viruses. In this paper, we focus on the periscope pipeline for analyzing ONT reads, due to its lightweight portable sequencing and, thus, potential for surveillance and bio-monitoring capabilities. For the ONT reads, periscope uses minimap2 (15) with default parameters (-ax map-ont -k 15 -w 10). Minimap2 maps the reads from a sample to a single reference representation of the viral sequence. After this, the resulting .bam file is processed in several steps. The first step retrieves the starting position of each read and, based on the starting position, assigns the reads to the corresponding amplicon using information on the amplicon positions, amplicon lengths, and primer positions provided by the ARTIC protocol (https://artic.network/ncov-2019/ncov2019-bioinformatics-sop.html). After the read has been assigned to an amplicon and the read start position on the amplicon is determined, the tool deduces if a read begins in an ORF using the known ORF positions given by a file with each amplicon’s starting and ending position. If the read is mapped with the start position inside the ORF, it is assigned to the ORF. The second step is to find the leader sequence in the mapped reads. To perform this task, periscope uses a local alignment approach using dynamic programming with biopython (22). The scoring parameters for the local alignment are +2 for a match, −2 for a mismatch, -10 for the gap opening penalty, and −0.1 penalty to extend the gap. If the read is assigned at an ORF, it is classified to the corresponding sgRNA with a label based on the leader’s alignment score. If the score exceeds 50, it’s categorized as HQ (High Quality). If the score falls within the range of 50 to a specified threshold, it’s classified as LQ (Low Quality). If the score is below the specified threshold, it receives the label LLQ (Low Low Quality). The reads not assigned to an ORF are labeled as non-canonical sgRNA if their leader score is HQ or LQ. All the amplicons that are not labeled as non-canonical sgRNA or sgRNA are labeled as genomic RNA (gRNA). Once the two-step-based read classification step is complete, statistics such as the proportion of each sgRNA are computed.

### Periscope-multi

We here present periscope_multi, a modified version of periscope. periscope_multi is implemented to preserve the same user interface, identical functionality, and output format to periscope. A schematic representation of the strategies used by periscope and periscope_multi can be found in Supplementary Figure S1. At a high level, periscope_multi uses multiple reference sequences representing the known variation of the sgRNAs of the genome under study. This multi-reference better represents the genomic variation that occurs and increases the length of the possible alignment between the read and references, which allows for more accurate and time-efficient analysis. Specifically, we made three design changes to the periscope pipeline.

**Figure 2.**
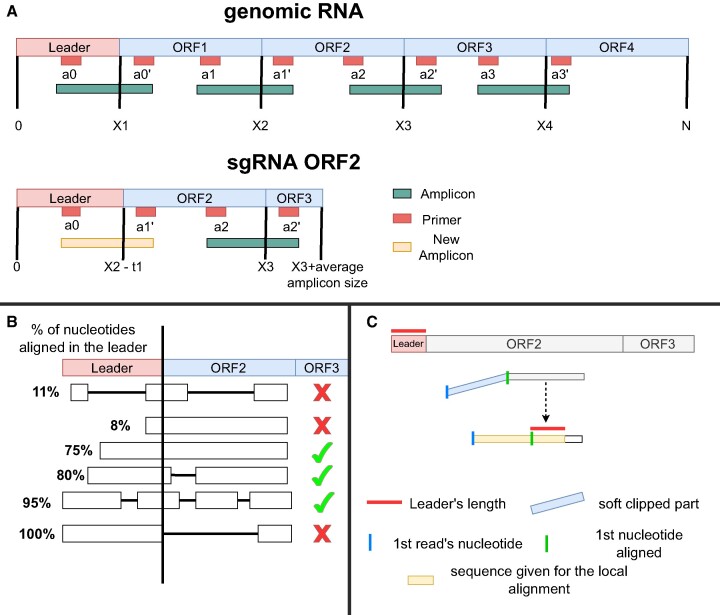
Panel **A** shows the construction of each sgRNA for the new reference in periscope_multi. The red segments represent the position of each pair of primers and the green bar are the resulting amplicons. The yellow segment shows the amplicon resulting from the new primer pair of *a*_0_ to $a_1^{\prime }$ due to the new location of each primer in the sgRNA. Panel **B** shows examples of the alignment needed for periscope_multi to consider a read as sgRNA. Panel **C** shows the local alignment in periscope_multi for three example reads. Only the soft-clipped part of the read plus the leader length is used. The corresponding yellow segments to the left show the sequence given for each of this reads.

First, periscope_multi creates a reference file with multiple sequences corresponding to the beginning of all the known sgRNA concatenated with the leader sequence, using the information available to periscope. Each sequence consists of the leader part, a given gene, and the start of the following downstream gene (see the sgRNA ORF2 in Figure 2A). Finally, the leader sequence is appended to the front of each sequence. Thus, the resulting length of our reference sequences will be the length of the gene (e.g. ORF2 in Figure 2A) plus the length of an amplicon. We also modified the S gene’s position to fit the position from previous work (23).

Secondly, periscope_multi, similar to periscope, uses minimap2 to perform read mapping to our multi-reference file. Periscope_multi then extracts the reference position of each mapped read. If the read aligns with more than a threshold *X* (*X* = 0.3) of the nucleotides in the leader sequence (approximately 8 nucleotides), periscope_multi labels the read as sgRNA (Figure 2B). Thus, unlike periscope, all the reads mapped to the beginning of the reference are labelled as HQ, avoiding the usage of the HQ, LQ and LLQ categories. In addition, less reads needs to be locally aligned to identify the leader sequence, as the leader is already aligned with the reference, speeding up the computational processing.

Thirdly, in order to further reduce the computational cost of periscope_multi, only the soft-clipped part of the read plus the leader length is used from the read to locally align against the leader sequence (Figure 2C). This simplification is based on the following reasoning. If the mapping is correct, the leader part has to be at the beginning of the alignment. Otherwise, that means the alignment is spurious, which means we discard it for further analysis. The local alignment score is then used to determine if the read is a non-canonical sgRNA, similarly to periscope. If the score is higher than a given threshold similar to periscope, the read is labeled as non-canonical sgRNA. Finally, periscope_multi converts back the positions of the reads with respect to the original genome provided both to periscope and periscope_multi.

### sgENERATE, a pipeline for evaluating ARCTIC-based methods

We developed a pipeline named sgENERATE to evaluate sgRNA prediction tools for the ARTIC protocol. sgENERATE, built in Python using snakemake (24), first generates datasets mirroring ARTIC data by extracting amplicon sequences from a reference using amplicon positions from ARTIC. We then simulate long-read sequencing data using pbsim2 (25), configuring parameters to match amplicon sizes (--length-mean 2000, --length-min 2000, --length-max 2000) and Nanopore error rates (--difference-ratio 23:31:46) corresponding to the ratio for substitutions, deletions, and insertions as Nanopore sequencing. The randomness for the --hmm_model model analysis is set with --seed 1234, and the R103 pbsim2 model mimics Nanopore sequencing. Users can adjust --accuracy-mean for error rates and -depth for sample size. A table with all the subgenomic RNA junction site from a study(23) on subgenomic RNA is used to reconstruct all the subgenomic RNA from the reference genome. This table is in .TSV format and can be modified by the users to fit another sgRNA format.

We determine chimeric primer pairs and resulting amplicons to mimic the proccess of an amplicon coming from an sgRNA product. In the sgRNA, leader primers are present, but the deletion sequence primers are missing, potentially creating new amplicons as shown in Figure 2A. We follow an assumption: a chimeric amplicon is built from a pair of primers, considering amplicon length and polymerization by the polymerase. Chimeric amplicons are generated using pbsim2, configured as before but with a maximum size 1.5 times the mean amplicon length. A final script merges amplicons to create a dataset. To mirror real data, the script randomly selects chimeric amplicons, ensuring they don’t exceed 1% of total reads. The result is a fastq file mimicking ARTIC protocol with primer scheme v3 (https://artic.network/resources/ncov/ncov-amplicon-v3.pdf).

Our pipeline has some simplifying assumptions compared to the ARTIC sequencing pipeline. Firstly, RT-PCR amplification errors are not simulated. Secondly, the reads do not contain a small sequence tag. The sequence tag distinguishes primers in different amplicon pools but can be removed using tools like TagCleaner (26). Nevertheless, our pipeline models the main steps and as we will show, can highlight several biases. In particular, the data generated by our pipeline can be seen as an easy dataset without noisy amplicons, and unexpected biological cases like the non-canonical sgRNA are not produced in our pipeline.

After generating the data, the pipeline runs benchmarked tools (in this study, periscope and periscope_multi) with the generated dataset. The benchmark result in six files: one fastq file with reads, two BAM files (alignments with raw reads and alignments with reads after classification to which amplicon they belong), and three CSV files (sgRNA count, normalized sgRNA count, and amplicon-sgRNA mapping). sgENERATE analyzes these files, providing graphical visualizations of sgRNA proportions and total reads. It also identifies common and unique sgRNAs between tools. Matplotlib (27) and matplotlib-venn construct the plots. sgENERATE is modular and can easily integrate additional tools that conform to the format of taking as input a .fastq file and providing as output the same CSV file as periscope and periscope_multi as well as an alignment file (.bam) with the same tag for the annotation of reads. Our evaluation pipeline can, therefore, be used to evaluate future bioinformatic pipelines for this sequencing protocol and aid the development of computational pipelines for analyzing such sequencing data. While previous studies (5,28) have compared tools like periscope (19), LeTRS (17), sgDI-tector (18) and CORONATATOR (29), no benchmark has generated or used simulated datasets that enable a ground truth to assess the tools’ capabilities in detail.

### Analysis and materials

We wanted to investigate whether periscope was accurate at (i) finding and quantifying sgRNA reads and (ii) finding non-canonical sgRNA reads. To validate (i), we simulated gold standard dataset using sgENERATE. To validate (ii), we used both simulated and biological reads from the periscope paper that consist of reads from nanopore sequencing using ARTIC protocol. For simulated data, the ground truth origin of reads is available. In this case, reads that are labeled as sgRNA by sgENERATE and assigned to the corresponding ground truth sgRNA by a tool are True Positives (TP), and reads that not labeled as sgRNA by sgENERATE but are predicted as sgRNA by the tools are False Positives (FP). Similarly, reads that are sgRNA but not predicted as such by the tools are False Negatives (FN), and reads that are not sgRNA and not predicted as sgRNA by the tools are True Negative (TN). The precision and recall is defined as $precision = \frac{TP}{TP+FP}$; $recall = \frac{TP}{TP+FN}$ . For the biological dataset, where ground truth is not available, we compared the performance, trend of the predictions (proportion of sgRNA and non-canonical sgRNA compared to simulations), and manually analyzed some of the aligned reads.

The simulated dataset, generated with sgENERATE and hereby referred to as the SIM dataset, consisted of 419 095 reads of median length 400 and average error rate 7%, where the fraction of sgRNA read was 1/100. We analyzed three biological datasets. The first one is the sample ERR5159329 which consists of 681 228 ONT reads sequenced with the ARTIC protocol. We hereby refer to this dataset as the BIO-SMALL dataset. The second one consists of 1145 samples of ONT reads with the project number PRJEB40972. This dataset consisted of a total of 305 338 021 reads and we refer to it as the BIO-LARGE dataset and are available on the European Nucleotide Archive (ENA; https://www.ebi.ac.uk/ena/browser/search). The details of each sample ID can be found in Additional File 1. This data is publicly available as part of the periscope study (19) and all the samples was generated using Oxford Nanopore Technology, more specifically a gridION system. The ARTIC protocol was used to amplify the viral samples, using the primer scheme V3. Finally, we also assessed the tools on Illumina data. The third dataset consists of 681,228 reads, using ARTIC protocol and sequenced with paired end Illumina protocol called BIO-SMALL-ILLUMINA. The dataset is accessible at ENA with the sample ID ERR5165937.

The SIM and BIO-SMALL datasets were analyzed on machine on Ubuntu 22.04.2 LTS with 32,0 GiB of RAM and a 11th Gen Intel^®^ Core^™^ i7-1185G7 @ 3.00GHz × 8 processor. The BIO-LARGE dataset was performed on a single cluster node running with Intel^™^ Xeon^™^ Gold 6130 CPU @ 2.10 GHz with 128 GB of RAM and Ubuntu 22.04. The performance comparisons are performed using the benchmark feature in snakemake. The leader sequence used in both periscope_multi and peri is 5’-AACCAACTTTCGATCTCTTGTAGATCTGTTCT-3’. As reference species we take the Wuhan reference genome available on the National National Center for Biotechnology Information available at (https://www.ncbi.nlm.nih.gov/) with the ID *MN908947.3*. The .gff file used to construct the multi-reference for periscope_multi was taken from the National Center for Biotechnology Information available at: https://www.ncbi.nlm.nih.gov/datasets/taxonomy/2901879/. Read visualization was performed using IGV (30). All figures are created using Python 3.9.13 and Matplotlib (27) 3.5.

## Results

### Periscope-multi accurately predicts sgRNAs

**Figure 3.**
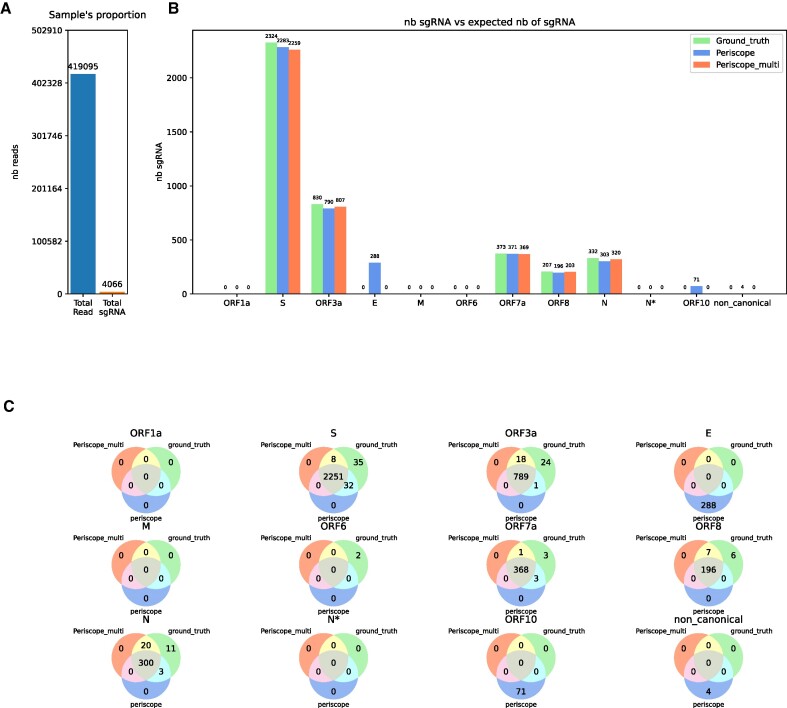
Result of periscope and periscope_multi on the SIM dataset from sgENERATE with an error rate of $7\%$. (**A**) Show the total number of read and the total number sgRNA introduce in the sample. (**B**) The number of sgRNAs found for each gene by the tools, the blue bar correspond to periscope, the orange to periscope_multi and the green one to the real number of sgRNA in the sample. (**C**) Venn diagrams showing the proportion of shared reads between periscope, periscope_multi and the ground truth.

We first analyzed periscope and periscope_multi, using all prediction classifications classes (HQ, LQ, LLQ). The SIM datasets show that periscope falsely detects sgRNA classified reads from E and ORF10 which are not in the sample (Figure 3B, C). Unlike periscope, periscope_multi does not produce any false classifications. However, in this configuration, periscope also finds slightly more TP than periscope_multi (Figure 3C).

**Figure 4.**
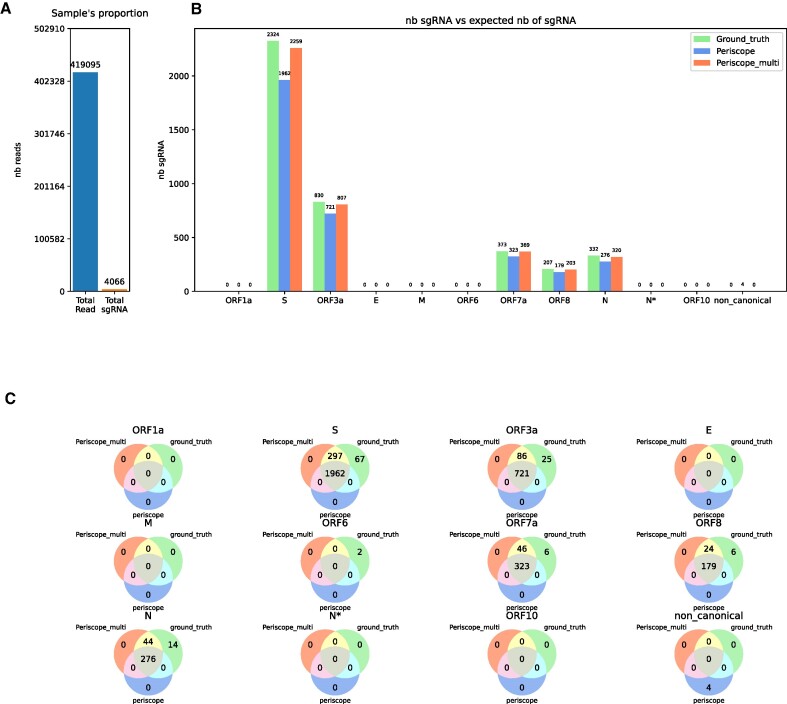
Result of periscope and periscope_multi on a simulated dataset from sgENERATE with an error rate of $7\%$ without the LLQ-labeled sgRNA for periscope. (**A**) Show the total number of read and the total number sgRNA introduce in the sample. (**B**) The number of sgRNAs found for each gene by the tools, the blue bar correspond to periscope, the orange to periscope_multi and the green one to the real number of sgRNA in the sample. (**C**) Venn diagrams showing the proportion of shared reads between periscope, periscope_multi and the ground truth.

We also investigated whether excluding the LLQ-tagged sgRNA reads improves periscope’s FP predictions in Figure 4. With this configuration, periscope no longer generates FP, but there is a sharp increase in the number of FN in all genes. Periscope underestimates the sgRNA from other genes. For example, it misses 364 sgRNA for S gene (15.65% of ground truth sgRNA from the S gene), 111 for ORF3a (13.3%), 52 for ORF7a (13.8%), 30 for ORF8 (14.35%) and 58 for N gene (17.3). In comparison, periscope_multi does not generate false positives and still allows us to find the majority of the sample’s sgRNAs with missing only 67 sgRNA for S gene (2.88% of ground truth sgRNA from the S gene), 25 for ORF3a (3%), 6 for ORF7a (1.6%), 6 for ORF8 (2.8%) and 14 for gene N (4.19). Figure 4 shows that in this configuration, periscope_multi outperforms periscope with a large number of correctly detected sgRNAs (Figure 4C).

In summary, periscope_multi has a precision and recall of 1.00 and 0.97, respectively, on SIM, while periscope has precision and recall of 0.92 and 0.97, respectively (with LLQ-labelled sgRNA) and 1.00 and 0.85 (without LLQ-labelled sgRNA). Thus, periscope_multi achieves beneficial sensitivity precision tradeoff.

**Figure 5.**
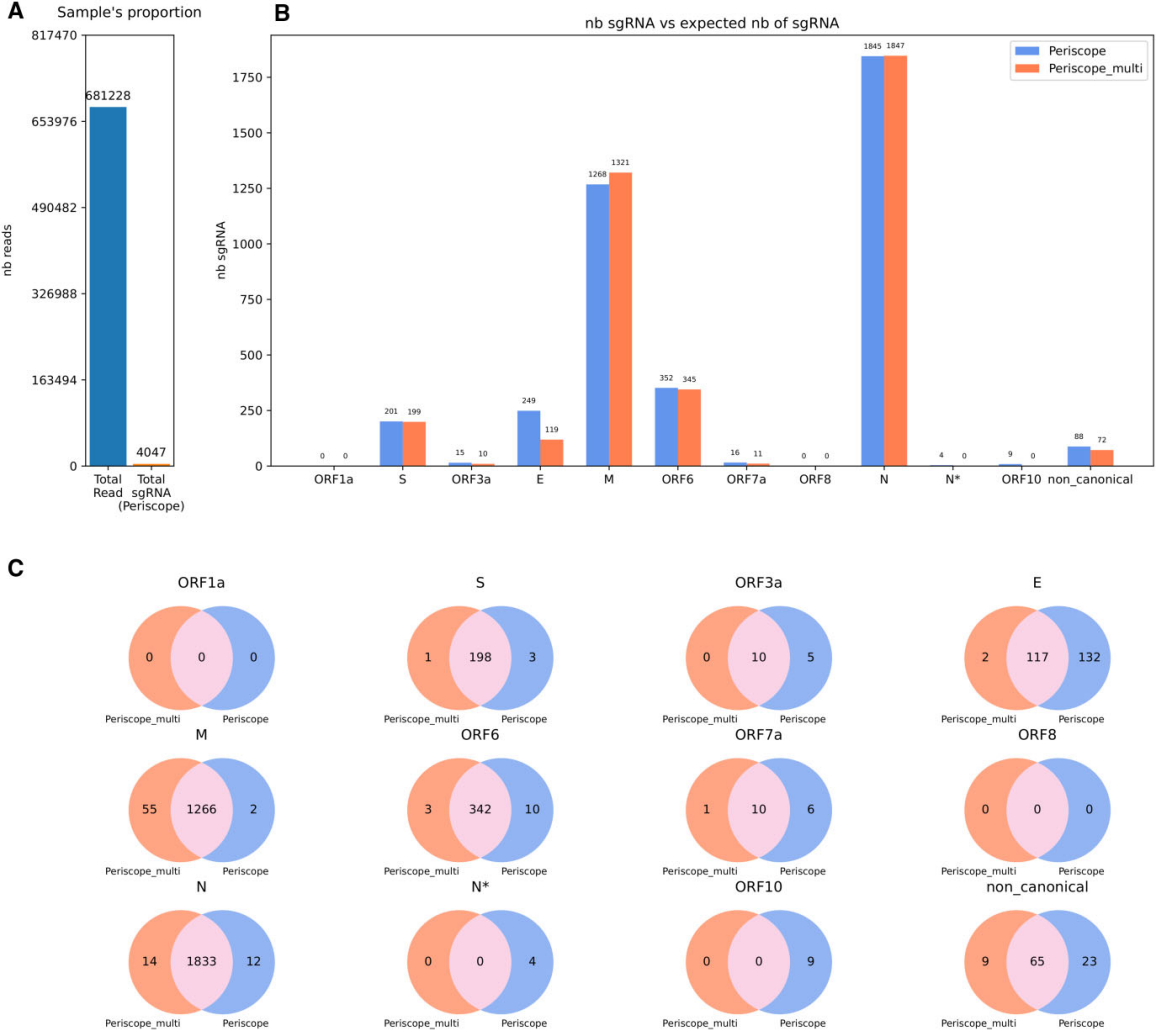
Result of periscope and periscope_multi on the BIO-SMALL dataset. (**A**) The total number of read and the total number of sgRNA found by periscope. (**B**) The number of sgRNAs found for each gene by the tools, the blue bar correspond to periscope and the orange to periscope_multi. (**C**) Venn diagrams showing the proportion of shared reads between periscope and periscope_multi.

As simulated data rarely models all the artifacts introduced in sequencing protocols, we investigated whether the overestimation (with LLQ-labelled sgRNA) was also present in biological data. When analyzing the BIO-SMALL dataset (Figure 5), we observed similar trends as in for the SIM dataset. The number of sgRNAs belonging to E is twice as high in periscope as in periscope_multi. There were also nine sgRNA belonging to ORF10 found only by periscope. For the other sgRNAs, the majority are shared between the two tools. When excluding the LLQ-labeled sgRNAs in periscope (Supplementary Figure S3), we see the same effects as with our simulated dataset. That is, a decrease in sgRNAs found by periscope and a harmonization of the results found by the two tools concerning E and ORF10.

### Additional tools and mapping configurations

In Supplementary Tables S1 and S2, we have also included results with other tools (sgDI-tector, LeTRS), read mappers (graphmap (31), BWA-MEM(32)), and configurations of minimap2 (details in Additional File 1). With more sensitive mapping parameters, periscope_multi finds slightly more reads ($< 1\%$ of total) categorized as sgRNA than when using default mapping parameters (Supplementary Tables S1 and S2, and Supplementary Figure S2). All the other tools or configurations give inferior results to periscope_multi.

### Soft clipping leads to an overestimation of non-canonical sgRNA

**Figure 6.**
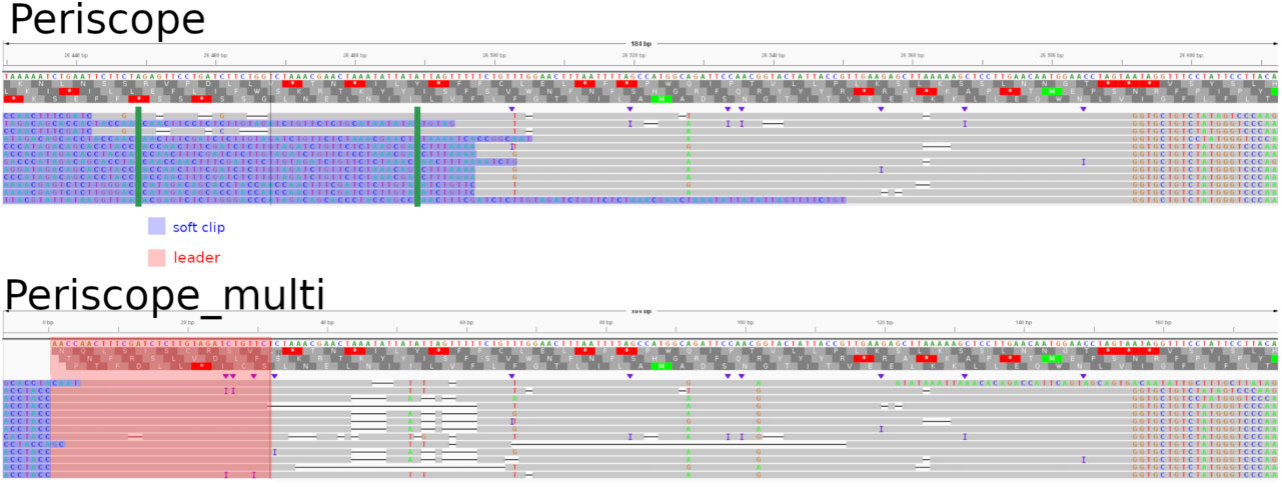
Picture of the different alignments obtained by periscope and periscope_multi Figure shows alignment of reads classified as non-canonical sgRNA by periscope and found as canonical by periscope_multi. The same 12 reads are present in the two pictures but there position differ.The region between the green bar represents ORF area which determines whether a read is subgenomic in periscope, and blue region shows soft-clipped part of the reads. A read alignment has to start in this region between green bars to be considered as a sgRNA, which is not the case for the reads aligned with periscope (upper panel). The red part shows the leader position in periscope_multi, which is softclipped in periscope’s alignments.

The absence of non-canonical sgRNA in the SIM dataset allows us to verify the presence of FP among the non-canonical sgRNAs. Figure 4 shows that periscope misclassified three reads as coming from non-canonical sgRNAs even with LLQ-tagged sgRNA reads excluded. As for the BIO-SMALL dataset, Figure 5 shows that periscope found 88 non-canonical sgRNAs while periscope_multi found 72 with the majority shared by the two tools (Figure 5C). However, 23 of the sgRNA are only found by periscope while only nine are only found by Periscope_multi. These 23 discordant predictions were split across several locations on the genome. We manually analyzed the region with the most discordant classified reads, consisting of 12 reads (for the M gene). Figure 6, shows results for 12 of the 23 non-canonical sgRNAs only found by periscope belonging in periscope_multi to S gene’s sgRNA. In this figure, alignment for periscope and periscope_multi using IGV (30) are shown.

The reads are aligned between the positions 26 430 and 26 612 in the SARS-COV-2 Wuhan reference genome. In the periscope_multi alignment, where the reference sequence corresponding to each sgRNA with the leader is included, we can see that the read is systematically mapped in the leader part (red area). However the aligned sequence following the leader includes several smaller or larger gaps. In Figure 6 when we compare the alignment positions given by periscope_multi with those given by periscope, we can see that except for one or two reads which have large alignment gaps after the leader, all the reads begin their alignment inside the ORF area and will be labeled as known sgRNA by periscope_multi. To study the detection performance for all sgRNAs simultaneously, additional datasets were simulated where all the canonical sgRNAs are in the sample at equal abundance in low proportions (details on the analysis are given in Additional File 1). We also performed these simulations with reads with varying error rates. It can be observed that, even though the error rate impacts periscope_multi, it remains the most effective tool, consistently achieving the highest F1 score (Supplementary Table S4).

### Reanalyzing the BIO-LARGE dataset reveals over detection of non-canonical sgRNA

**Figure 7.**
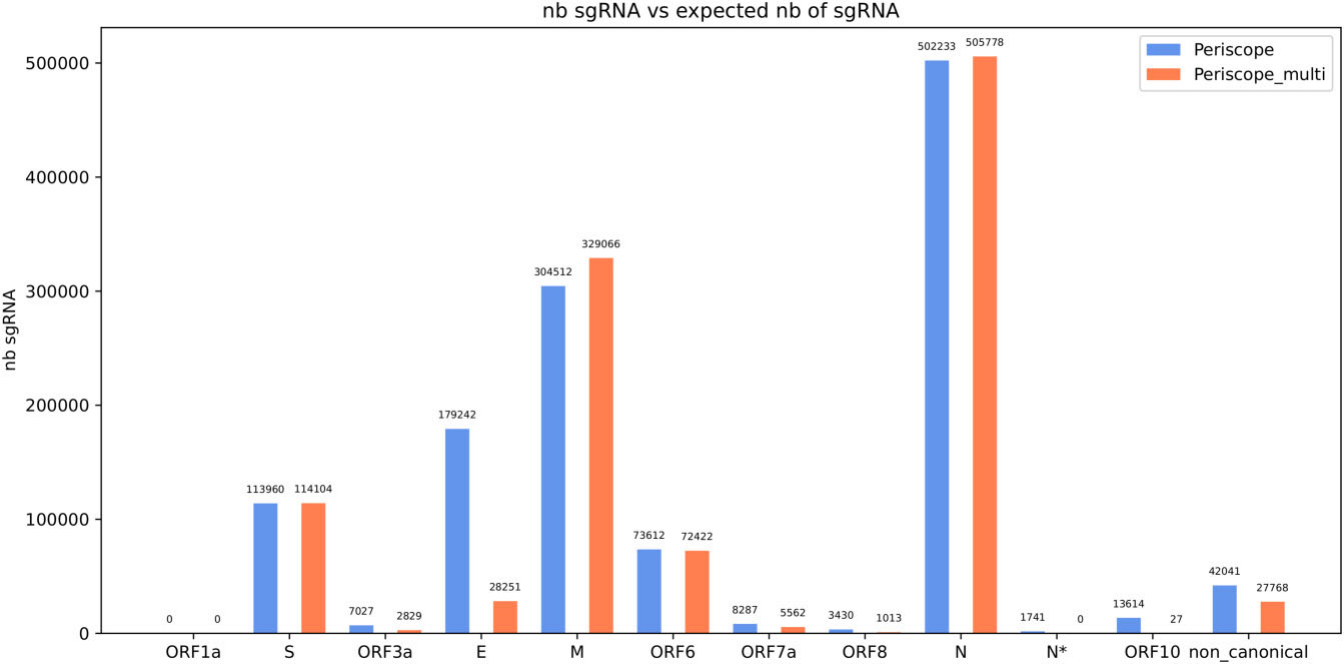
Result of periscope and periscope_multi with the BIO-LARGE dataset the bar represent of sgRNA found in all the sample, the blue bar correspond to periscope result and the orange to periscope_multi

To confirm the results shown using the SIM-dataset and the BIO-SMALL dataset, we evaluated periscope_multi and periscope (using LLQ-labelled sgRNA) on the BIO-LARGE dataset (Figure 7). We observe that, similarly to previous experiments, periscope finds substantially more sgRNAs for the E and ORF10 genes than periscope_multi. Supplementary Figure S4 shows that, as in the simulated dataset, if the LLQ-labeled are discarded, the same proportion of sgRNA are found in the two tools, but decrease the overall number of sgRNA found by periscope for the other gene. For the M, N, ORF6 and S genes periscope_multi found slightly more sgRNA than periscope. For all the other genes, periscope finds more sgRNAs than periscope_multi. In addition, periscope found 42 041 non-canonical sgRNA, a 51% higher estimation compared to periscope_multi’s 27 768 non-canonical sgRNA classifications.

**Figure 8.**
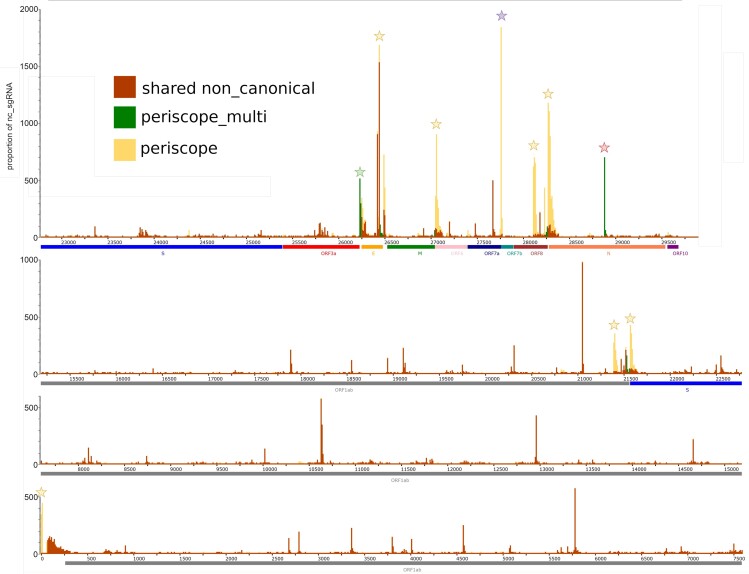
Positions and proportion of the non-canonical sgRNA among the COVID-19 genome from the results of the BIO-LARGE dataset. In the figure, all the non-canonical RNA are bucketed according to their position (10 nucleotide windows). The height of the spike illustrates the number of non-canonical sgRNA in the windows. The height corresponds to the total number of sgRNAs in a window is divided by 10. The red bar shows that in this window, the two tools share the same number of sgRNA. The green bar shows non-canonical sgRNA found only by periscope_multi, and the yellow represents those found only by periscope. The star represents an area with a high divergence between the two tools. Yellow stars display a divergence from periscope. Green stars represent divergence with periscope_multi, and the red stars show divergence with periscope_multi corresponding to the sgRNA labeled as N* in periscope, and the blue star shows divergence with periscope corresponding to ORF7b that are considered as canonical sgRNA in periscope_multi and not in periscope. Below the figure are the genes annotated according to their position.

We studied the locations of the non-canonical sgRNA predictions more closely. Figure 8 shows a representation of all the sgRNA positions on the SARS-COV-2 genome. There are many high abundance non-canonical prediction sites where periscope and periscope_multi agree (red peaks). However, as can be seen in yellow peaks in Figure 8, periscope predicts several high abundance peaks of non-canonical sgRNA that periscope_multi does not predict. Some notable discrepancies are marked with stars. For example, for the E gene, periscope_multi finds a high amount of sgRNAs at one position while periscope’s predictions at the site are more spread out.

Two peaks (indicated by red and blue stars) likely arise from the distinct gene classification systems used in periscope_multi and periscope. In the periscope system, there is an additional canonical sgRNA called N*, which corresponds to the same position as the non-canonical sgRNA identified by periscope_multi under the red star. Similarly, in periscope_multi, there is an extra canonical sgRNA named ORF7b, sharing the same position as the non-canonical sgRNA marked by periscope with the blue star. Overall, we see that periscope_multi non-canonical sgRNA predictions are much fewer and, e.g. for the E gene, more precise.

### Validating non-canonical sgRNAs in BIO-LARGE

An interesting aspect of non-canonical sgRNAs is if they are coding for the same proteins as canonical sgRNAs or whether the proteins get truncated. We wanted to assess this for all periscope_multi’s non-canonical sgRNA predictions from the BIO-LARGE dataset. We took all the 27 768 reads classified as non-canonical sgRNA by periscope_multi and inferred the protein they coded for using a consensus approach of four alignment strategies to find the position in the genome where the non-canonical sgRNA most likely starts (details on the analysis are given in Additional File 1). We couldn’t find a high-confidence consensus start position for all the non-canonical sgRNAs, so we only consider two high-confidence cases; either all four, or three out of the four alignments agree on a position. We then use ORFfinder (33) to extract the Open Reading Frames (ORFs) from the reference sequence at the start position of the non-canonical sgRNA alignments, and compare them with the ORF of the canonical protein. For 16 037 reads (57.8% of total) all four alignments agree, with 9466 of them (59%) normal proteins and 6571 of them (41%) truncated. In the case where three alignments agree, 4632 reads (16.6% of total) are found with 2688 (58%) of them normal proteins and 1944 (42%) of them truncated. The 7099 (25.6%) remaining reads are labelled as uncertain in the protein columns. A CSV file with all the non-canonical sgRNA reads and whether they code for the same protein as the sgRNA from the same gene or not is available as Additional File 2.

### Predictions on BIO-SMALL-ILLUMINA

We also tested periscope_multi on the BIO-SMALL-ILLUMINA dataset (Supplementary Table S3 and Supplementary Figure S7). We observe that even though periscope_multi is not designed for Illumina sequencing reads, it can retrieve more sgRNA reads than periscope. However, tools like sgDI-tector, that are designed for Illumina reads, find more sgRNAs than both periscope and periscope_multi and are recommended for Illumina data.

### Runtime and memory usage

We monitored the computational time and memory usage of periscope and periscope_multi on all the 1145 samples in BIO-LARGE (Supplementary Figures S5 and S6) ordered by increasing resources by periscope_multi. We observe a large fluctuation in runtime per instance for periscope. In contrast, periscope_multi is more stable across samples and periscope_multi is more than three times faster than periscope with a total CPU time of 3.4h compared to 11.0h for periscope.

## Discussion

We implemented an evaluation pipeline sgENERATE that simulates ARTIC-protocol data and used it to compare periscope and periscope_multi for detecting sgRNA. We compared the results between the SIM-dataset, BIO-SMALL and BIO-LARGE datasets. While the release of periscope is a useful tool for the study of sgRNAs, we conclude that periscope overestimates some of the sgRNA and falsely calls some of the non-canonical sgRNA. We were able to correct much of this bias in our modified version periscope_multi. The experiments also shown us that our pipeline sgENERATE have been able to simulate data that highlights problematic cases in real datasets, and can provide dataset with ground truth allowing to compare further tools for sgRNA detection.

### Periscope bias and minimap2 limitation

As it could be seen in Figures 3, periscope overestimates the number of the E and ORF10 sgRNA, which likely occurs also in biological data (Figures 5 and 7). The analysis in the periscope paper didn’t include the LLQ-labeled sgRNA. However, if LLQ-labeled sgRNA is excluded, periscope underestimate the number of the sgRNA (Figure 4, and Supplementary Figures S2, S3). For example, in Figure 4 almost 15% of the total number of sgRNA are missing for ORF7a. This can be explained by the way periscope determines if a read is subgenomic. The reads starting within the ORF area (between green bars in Figure 6) are considered subgenomic for the corresponding gene regardless of the leader’s presence. The leader is only used to establish a score for category classification (i.e., HQ, LQ or LLQ), so periscope can’t distinguish a read with a noisy leader from a read without the leader. In the periscope_multi approach, the leader should be mapped to the reference for a read to be considered subgenomic. This allows the tool to distinguish a noisy leader sequence and a random sequence of the reference genome. Periscope_multi will, therefore, be able to estimate the number of sgRNAs better.

Figure 3 shows that periscope make false positive non-canonical sgRNA, and according to Figures 5 and 6, a big part of the non-canonical sgRNA called by periscope is due to minimap2’s excessive soft clipping of the read, making the alignment start further downstream on the reference than what is considered correct. It means that the strategies of periscope will highly depend on the quality of the sequencing data. Consequently, the divergence of the non-canonical sgRNA seen in Figures 7 and 8 are likely due to a misclassification by periscope. That will lead to an overestimation of the number of non-canonical sgRNA. However, we can see that adding the leader in the reference, as in periscope_multi, improves the estimation of the non-canonical sgRNA. This addition increases the length of the potential region to align, which is critical for the seed-and-chaining function of minimap2 (34), which can use the seeds from the leader to avoid excessive soft-clipping. Periscope_multi strategies allow to obtain a less noisy visualization of the non-canonical sgRNA, but Figure 8 shows that even if a big part of the non-canonical sgRNA is discarded, some of them stay present. This can be observed for the E and M genes. Further study needs to be done to understand whether these reads are false positives.

In the case of ORF7b, there is uncertainty. A study (35) claims that ORF7b does not produce its own sgRNA but is produced from ORF7 sgRNA mRNA. However, int the results from the BIO-LARGE dataset a significant number of sgRNAs for ORF7b are found. Regarding the ORF10 sgRNA, periscope identifies only 12 sgRNAs if the LLQ-labelled are excluded. Although periscope_multi detects slightly more ORF10 sgRNAs (27), we share the conclusions of (19) that no clear assertion can be made regarding the presence of subgenomic RNA for the ORF10 gene with this dataset.

### Other species with sgRNA

In our study, we focus on SARS-COV-2, but other species with sgRNA exist such as MERS-COV, SARS-COV-1, and other coronaviridae, e.g. OC43 (36). In addition, a long list of positive-strand viruses exist, and share the mechanism of sgRNA synthesis (36). Due to the lack of existing ARTIC long-read datasets, we could not compare periscope and periscope_multi on other species. Moreover, both periscope and periscope_multi require the positions of the genes and the leader sequence. If this information is unknown for a species, new tools for discovering sgRNA will be needed.

### Future work

We here focused on the algorithm design of periscope and ways to improve it using a multi-reference approach, periscope_multi. To fairly quantify periscope_multi’s improvement over periscope, we kept the alignment parameters identical to periscope for the main analysis. While we tried non-default alignment options and other alignment alternatives (Supplementary Tables S1–S3), we believe that alignment methods designed for long-read ARCTIC type data can improve prediction. The ARTIC protocol dataset has errors both from the amplification and sequencing while being shorter than typical ONT reads. Moreover the detection of non-canonical subgenomic RNAs is still based on the detection of the leader sequence, which can sometimes be missing or of poor quality. ESPRESSO (37), a tool for transcript detection, uses high-quality spliced reads as a reference to realign and correct other reads. Applying such methods to non-canonical subgenomic RNAs could reduce the number of false positives among non-canonical sgRNAs and also enable the recovery of certain non-canonical sgRNAs with a partial or noisy leader sequence. In addition, viral sequences have less repetitive sequences than eukaryotes and prokaryotes. In contrast, with improving error rates of long reads (38), the development of mapping tools has mainly focused on rapid mapping of longer high-quality reads to larger repetitive references (34,39,40). The ARTIC protocol would need development in the opposite direction, i.e., short and high error rate reads with frequent indels. For example, in our ORF prediction analysis of non-canonical sgRNAs, over 25% of non-canonical sgRNAs did not have a solid consensus start position of the alignment when aligned with different approaches. Fast and indel tolerant alignment methods have been designed for short reads (41), and sensitive but very slow alignment methods exits (42). Nevertheless, developing mapping tools specifically designed to rapidly and accurately align viral RNA will improve bioinformatic pipelines, which make decisions based on alignment scores.

## Conclusion

Although viral organisms are well studied, most bioinformatics methods that study viruses are based on computational tools built for eukaryotes (43,44). This study presents a bioinformatic evaluation pipeline, sgENERATE, and a tool for accurate sgRNA detection, periscope_multi. sgENERATE simulate data from ARTIC protocol and helps assess the accuracy of sgRNA prediction tools. Using sgENERATE we showed that the state-of-the-art method periscope is biased by over-estimating the number of some sgRNAs, and by falsely finding non-canonical RNAs. Guided by sgENERATE we improve periscope’s informatics pipeline to produce more accurate canonical and non-canonical sgRNA predictions while having over three times faster runtime.

## Supplementary Material

gkae687_Supplemental_Files

## Data Availability

sgENERATE and periscope_multi are available at https://github.com/ThomasBaudeau/sgENERATE (https://doi.org/10.6084/m9.figshare.25343137.v1) and https://github.com/ThomasBaudeau/periscope_multifasta (https://doi.org/10.6084/m9.figshare.25343140.v1), respectively.
