# Supplementary material for: Improved sub-genomic RNA prediction with the ARTIC protocol
Source: Nucleic Acids Res. 2024 Aug 16;52(17):e82. doi: 10.1093/nar/gkae687 (PMC11417393; doi:10.1093/nar/gkae687)
Supplement: gkae687_Supplemental_Files [file gkae687_supplemental_files.zip › Additional_File_1.pdf]

Published online DD MM YYYY *Nucleic Acids Research, YYYY, Vol. xx, No. xx* ***1–10***
doi:10.1093/nar/gkn000

Additional File 1: Improved sub-genomic RNA prediction with the ARTIC protocol

Thomas Baudeau [1,*], Kristoffer Sahlin [2,*]

[1]Univ. Lille, CNRS, Centrale Lille, UMR 9189 CRIStAL, F-59000, Lille, France
[2]Department of Mathematics, Science for Life Laboratory, Stockholm University, 106 91, Stockholm, Sweden

ADDITIONAL RESULTS

Additional datasets and read errors rates

We also simulated reads at various error rates and with an equal abundance of all sgRNA types to better illustrate the classification performance across different sgRNA types. Supplementary Table S4 shows the result of `periscope_multi` and `periscope` in both settings on datasets with mean error rates of 4%, 7%, 10%, 15%, and 20%. In all five datasets, a fixed quantity of 100 reads were simulated from each sgRNA type. This corresponds to a rough abundance rate of 0.02% for each sgRNA type compared to the total number of reads generated by pbsim2 for the sample (between 415,567 and 500,135 reads for each dataset due to coverage variability in pbsim2). The datasets contain no non-canonical sgRNAs. The experiments show results similar to those observed in Figures 3 and 4, indicating that while `periscope` detects slightly more sgRNAs than `periscope_multi` , it has a very high false negative rate for some types, leading to a significant drop in the F1_score. We note a slight bias in `periscope_multi` , which incorrectly considers certain sgRNAs of the E gene as non-canonical. This bias can also be seen in Figure 8, with a higher proportion of E sgRNAs just after the E gene. Finally, the results show that, as expected, both tools are sensitive to error rates, as seen by the F1 score decreasing with higher error rates. However, `periscope_multi` obtains the best F1_score across all error rates.

Short read specificity

Additional Table S3 shows the result of the tool on the BIO-SMALL-ILLUMINA dataset. sgDI-Tector find between 10% and 50% more sgRNA than periscope and periscope_multi. LeTRS found slightly more sgRNA than periscope_multi (less than 10, except for ORF6). Periscope found the fewest sgRNA. To confirm the additional sgRNAs found by periscope_multi, we use IGV to compare the alignments (Additional figure S7). The differences are due to some reads that are mapped by BWA-MEM to the leader region, i.e., the first 60 nucleotides in the reference genome. This happens because the leader part of the read is larger than the short segment of the gene present in the read. We also observed occasional short reads (less than 1%) were classified as sgRNA by periscope_multi. We believe they are misaligned because they contained several errors in the part aligning to the leader. Finally, there are fifteen reads only found by periscope and not by periscope_multi. These are due to read only containing small parts of the leader region and, thus, are discarded by periscope_multi due to the threshold of a minimum of 30% of the nucleotides of the leaders needed for a read to be considered as non-canonical.

There is no graphical comparison between periscope, periscope_multi and the other tools because LeTRS and sgDI-tector didn't provide from where read each sgRNA come from. Moreover, we lack ground truth for the BIO-SMALL-ILLUMINA dataset, and thus, we can not be sure about the result. In (1), the authors claim that sg-DI(2) are better for detecting sgRNAs with short reads, which is in line with our results.

Additional tested tools and configurations

In addition to benchmarking `periscope` and `periscope_multi` using minimap2 with default parameters which constitutes the main evaluation, we included other combinations of methods described here. We include:

- The two tools LeTRS and sgDI-tector.
- Graphmap (3) as an aligner for `periscope` .
- `periscope_multi` with BWA-MEM (4)
- `periscope_multi` using minimap2 with parameters -w 1 and -k 10 (default -w 10 and -k 15), which we denote `periscope_multi` (tuned). This parameter setting allows for more accurate read mapping but increases runtime and memory usage.

*To whom correspondence should be addressed. ksahlin@math.su.se, thomas.baudeau@univ-lille.fr

© YYYY The Author(s)
This is an Open Access article distributed under the terms of the Creative Commons Attribution Non-Commercial License (http://creativecommons.org/licenses/by-nc/2.0/uk/) which permits unrestricted non-commercial use, distribution, and reproduction in any medium, provided the original work is properly cited.

Graphmap does not support multiple references and thus is not compatible with `periscope_multi`. For BWA-MEM with `periscope_multi`, many reads are not correctly placed on the genome. This is likely because BWA-MEMs alignment profile is adapted for Illumina short-read alignments to mammalian-sized genomes.

Finding truncated proteins in BIO-LARGE

To construct a database of non-canonical sgRNAs and the effect on the coding protein, we performed the following analysis. We first re-aligned all reads classified as non-canonical sgRNA in four different modes; (1) minimap2 in splice mode aligned to the SARS-COV-2 genome, (2) minimap2 in the same configuration than `periscope_multi`, (3) minimap2 default parameters but without the leader part and with only the first 200 nucleotides of the read aligned to the multiple reference, and (4) minimap2 default parameters but without the leader part and the soft-clipped at 5’ end aligned to the multiple reference. We used these four settings because the mode (1) with splicing can detect chimeric read or spurious alignment. (2) is our reference alignment, (3) is to ensure that the starting position of the alignment is correct. Indeed, if we consider a sequence A containing two sub-sequences Ab and Ac from two distinct regions of the genome, it is highly likely that the mapper will choose the position of the longer sub-sequence as the starting position for the alignment. This analysis therefore allows us to look at the starting position of the 1st sub-sequence. (4) allows us to discard possible negative interaction with primer of the read. We then designed a python script to compare for each read the four alignments produced under the different settings. We produce consensus positions from the alignments as follows with the help of a python script we collect the start position on the reference of each alignment and use as consensus position the position extracted on the reference when we have at least 3 alignment with the same position. The sequence in the genome corresponding to the positions of the consensus alignments is extracted. We then use ORFfinder(5) to extract the Open Reading Frames (ORFs) from the reference sequence. We use the reference sequence to overcome any read errors that would break the ORFs. For each found ORF, we compare them to the first ORF found in the corresponding canonical sgRNA. We create a CSV file of reads and indicate whether they code for a normal or truncated protein relative to the position found by ORFFinder for each gene. The genes used as references are extracted from NCBI under the gene IDs: 43740578; 43740568; 43740569; 43740570; 43740571; 43740572; 43730574; 43740575; 43740576; 43740577. This file is available as additional file 2.

ADDITIONAL TABLES

Tool \ sgRNA	S	ORF3a	E	M	ORF6	ORF7a	ORF8	N	ORF10	nc-sgRNA
Ground_truth	2334	830	0	0	0	373	207	332	0	0
periscope_multi (tuned)	2273	827	0	0	0	369	207	326	0	0
periscope_multi	2259	807	0	0	0	369	203	320	0	0
periscope (without LLQ)	1962	721	0	0	0	323	179	276	0	4
LeTRS	1507	538	0	0	0	269	119	211	0	0
Periscope	2283	790	288	0	0	371	196	303	71	4
periscope_multi (BWA-MEM)	2056	699	0	0	0	336	181	295	0	184
periscope (graphmap)	324	699	0	0	0	313	102	21	0	2053
sgDI-tector	0	0	0	0	11	138	155	3	0	8

Table S1. Results for the SIM dataset. Each column represent the number of sgRNAs found by the tools. The rows are sorted in descending order of accuracy with the most favourable result appearing in the first row.

Tool \ sgRNA	S	ORF3a	E	M	ORF6	ORF7a	ORF8	N	ORF10	nc-sgRNA
periscope_multi (tuned)	199	10	125	1341	346	11	0	1857	0	67
periscope_multi	199	10	119	1321	345	11	0	1847	0	72
periscope (without LLQ)	193	10	122	1244	336	10	0	1798	0	88
LeTRS	187	10	111	1159	319	11	0	1619	0	/
periscope_multi (BWA-MEM)	189	9	71	1304	277	11	0	1802	0	428
Periscope	201	15	249	1268	352	16	0	1845	9	88
periscope (graphmap)	21	3	10	107	3	1	0	10	0	11743
sgDI-tector	0	1	0	17	0	0	0	11	0	0

Table S2. Results for the BIO-SMALL dataset. Each column represent the number of sgRNAs found by the tools.

Tool \ sgRNA	S	ORF3a	E	M	ORF6	ORF7a	ORF8	N	ORF10	nc-sgRNA
sgDI-tector	63	364	41	161	104	190	65	510	0	50
LeTRS	43	256	26	115	78	159	43	408	1	/
periscope_multi (BWA-MEM)	43	246	20	115	42	147	56	407	0	51
Periscope	34	239	7	109	13	140	36	340	9	38

Table S3. Table of the result of all the tools for BIO-SMALL-ILLUMINA dataset. Each row and column represent the number of sgRNA found by one of the tool.

ER	Tool name	S	ORF3a	E	M	ORF6	ORF7a	ORF8	N	ORF10	nc_sgRNA	F1_score
-	ground thruth	100	100	100	100	100	100	100	100	100	0	1
4	pericope	100	98	242	100	98	98	97	96	124	0	0.90
4	periscope without LLQ	92	93	97	98	95	97	92	91	93	0	0.97
4	periscope multi	98	98	93	100	98	97	96	95	97	2	**0.98**
7	pericope	100	98	271	100	99	98	97	97	141	0	0.88
7	periscope without LLQ	88	85	93	92	95	91	91	93	94	0	0.95
7	periscope multi	98	98	93	100	98	97	96	95	97	2	**0.98**
10	pericope	96	90	615	93	97	98	88	85	199	4	0.70
10	periscope without LLQ	72	78	80	70	72	67	71	67	75	4	0.83
10	periscope multi	93	92	82	91	89	97	89	87	88	5	**0.94**
15	pericope	90	75	1342	87	84	93	80	58	469	5	0.43
15	periscope without LLQ	40	55	42	43	44	45	46	31	52	5	0.61
15	periscope multi	88	72	33	81	69	83	77	57	73	12	**0.81**
20	pericope	80	57	1534	73	71	64	43	51	753	4	0.29
20	periscope without LLQ	22	19	16	31	31	17	20	18	27	4	0.36
20	periscope multi	61	43	14	63	51	55	42	40	52	6	**0.63**

Table S4. Table of the result of Periscope (with and without LLQ labelled sgRNA) and Periscope_multi for customs datasets with error rate (ER) of 7, 10, 15 and 20 and with 100 sgRNA for all the sgRNA possible. Boldfaced values indicate the highest F1_score.

ADDITIONAL FIGURES

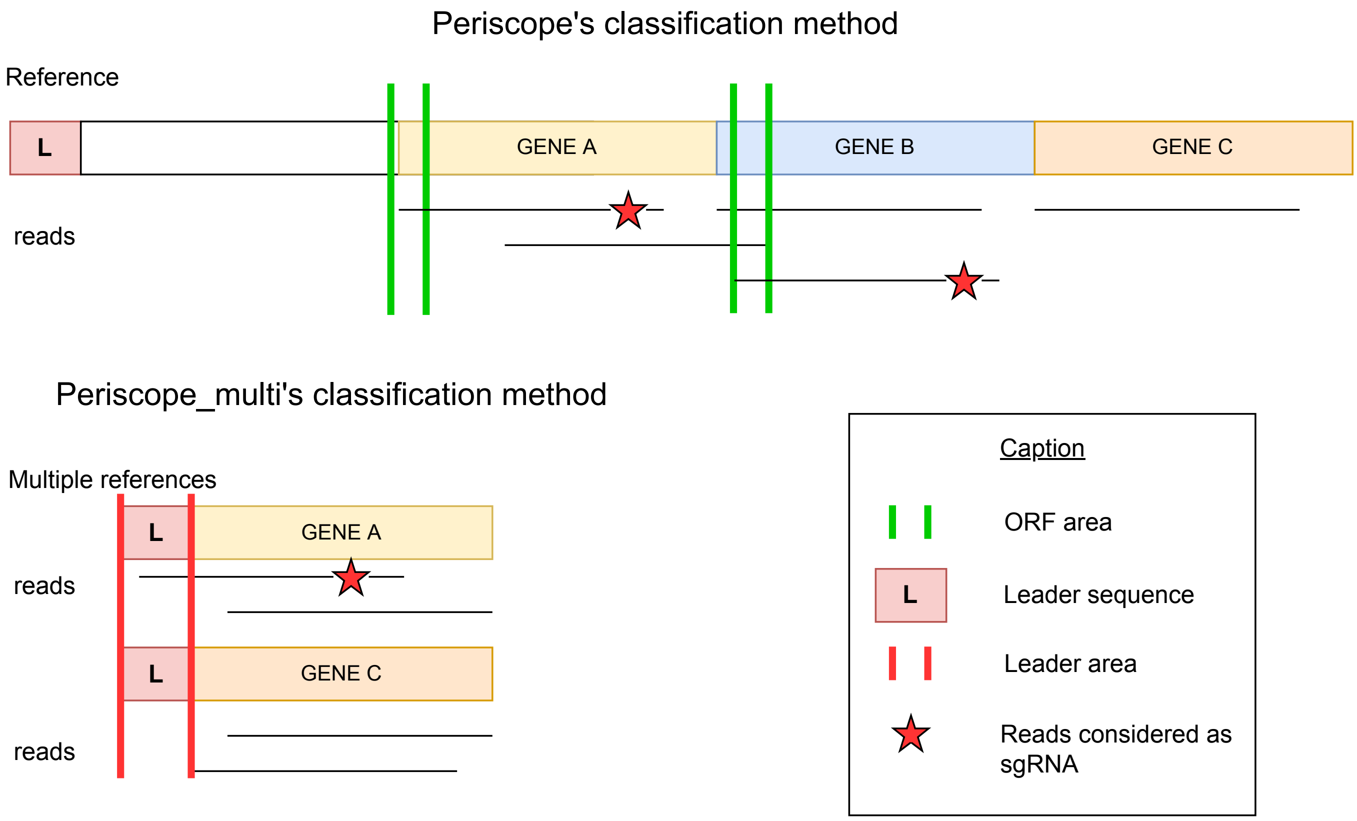

Figure S1. Schematic representation of the sgRNA classification methods between `periscope` and `periscope_multi`. The green area represent the begin and end of an ORF. In `periscope` a read is considered as sgRNA when it start in this area. The read considered as sgRNA as labelled with a red star. In `periscope_multi` a read is considered as sgRNA when a read is aligned in the leader area.

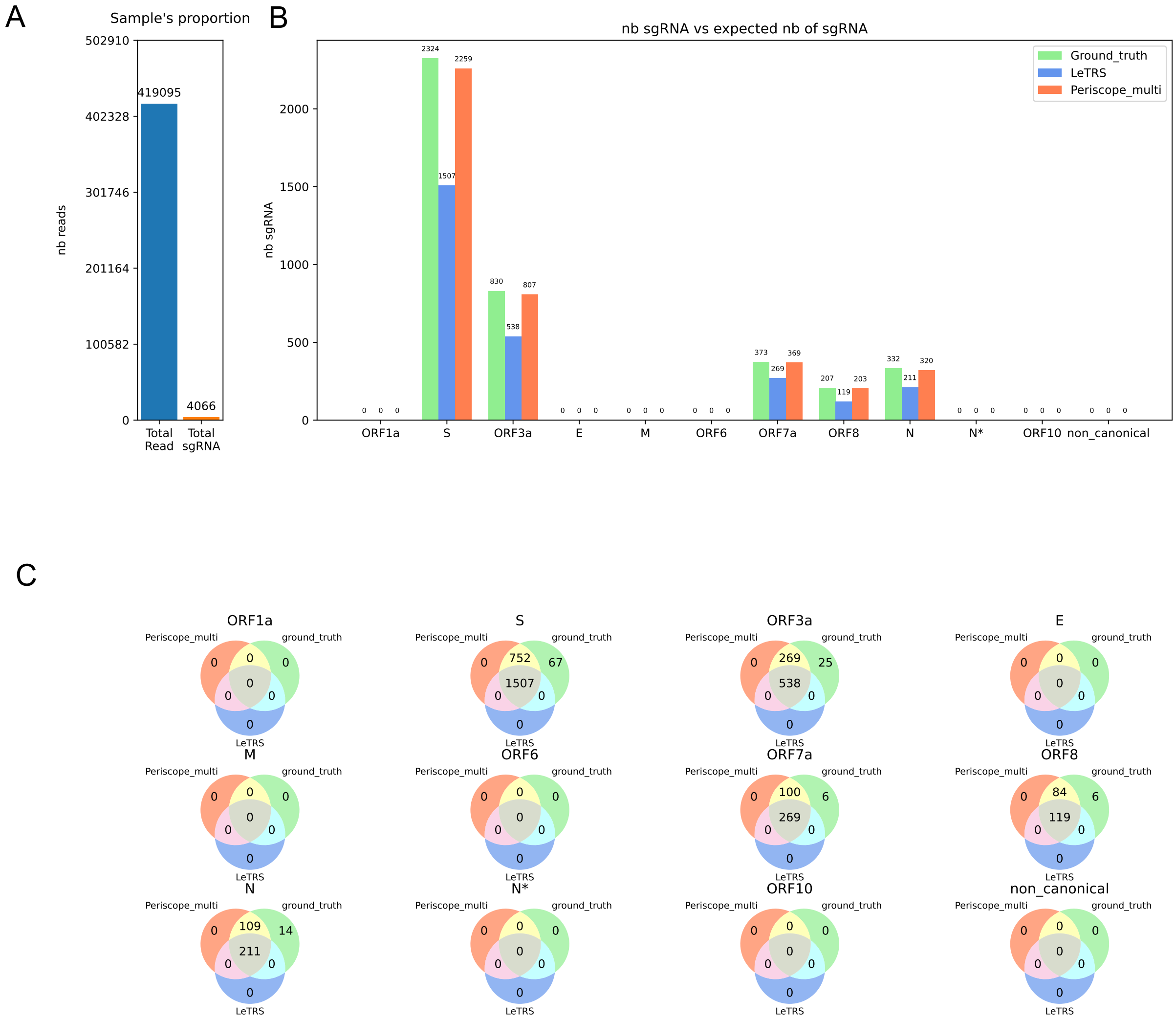

Figure S2. Result of LeTRS and `periscope_multi` on the SIM dataset A) Show the total number of read and the total number of sgRNA. **B)** Show the numbers of sgRNA found for each gene by the tools, the blue bar correspond to LeTRS and the orange to `periscope_multi` . **C)** are Venn diagrams showing the proportion of shared reads between `periscope` and `periscope_multi`

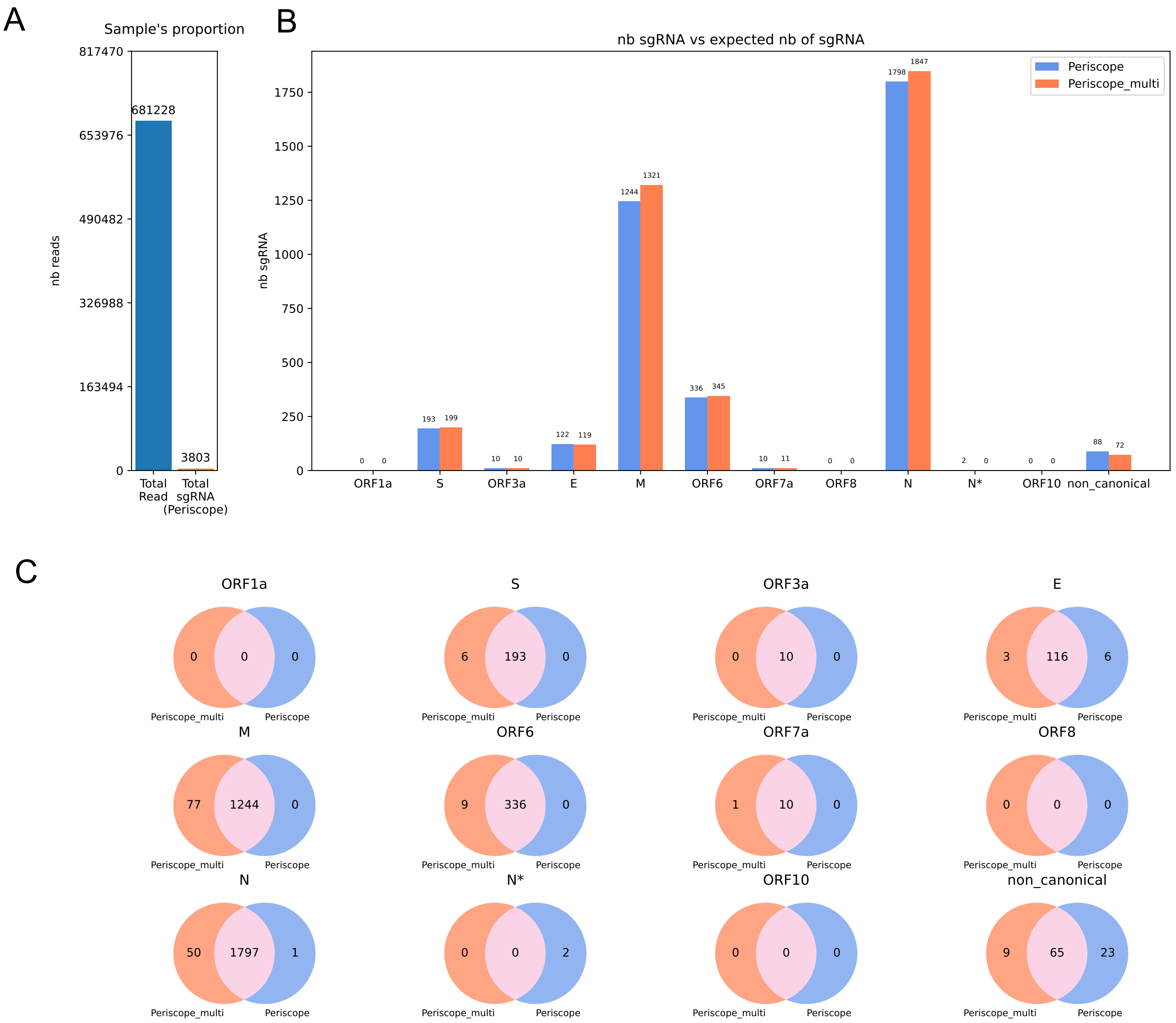

Figure S3. Result of `periscope` and `periscope_multi` on the BIO-SMALL dataset without the LLQ-labelled sgRNA A) Show the total number of read and the total number of sgRNA found by `periscope` . **B)** Show the numbers of sgRNA found for each gene by the tools, the blue bar correspond to `periscope` and the orange to `periscope_multi` . **C)** are Venn diagrams showing the proportion of shared reads between `periscope` and `periscope_multi`

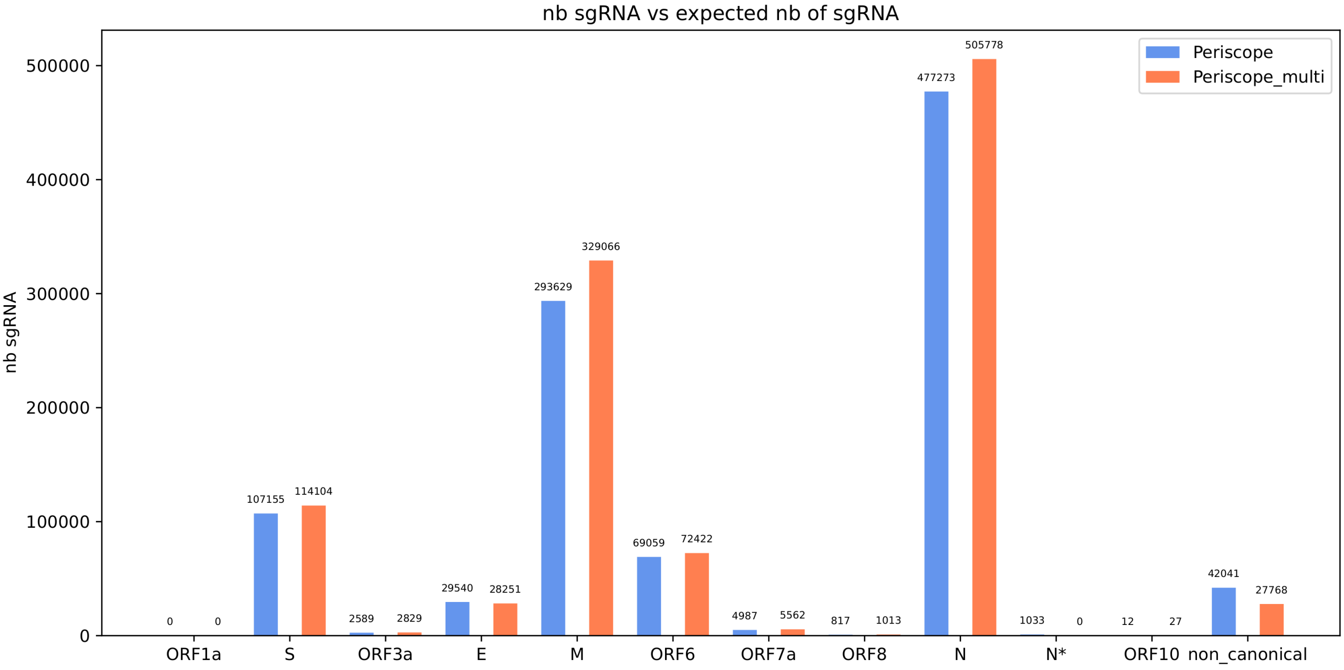

Figure S4. Result of `periscope` and `periscope_multi` with the BIO-large dataset without the LLQ-labelled sgRNA The bar represent of sgRNA found in all the sample, the blue bar correspond to `periscope` result and the orange to `periscope_multi` .

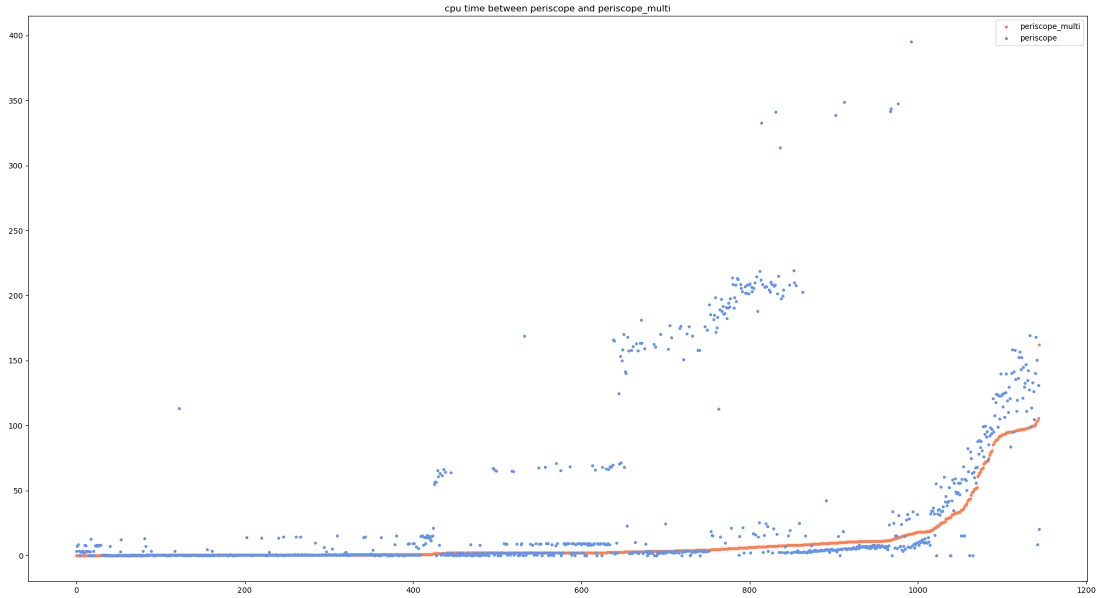

Figure S5. CPU time of `periscope` and `periscope_multi` for each sample of the BIO-large dataset The blue point correspond to `periscope` result and the orange to `periscope_multi` . Each point represent the cpu time for one of the sample of the Bio-large dataset. The samples are sorted in ascending order of time spent with `periscope_multi`

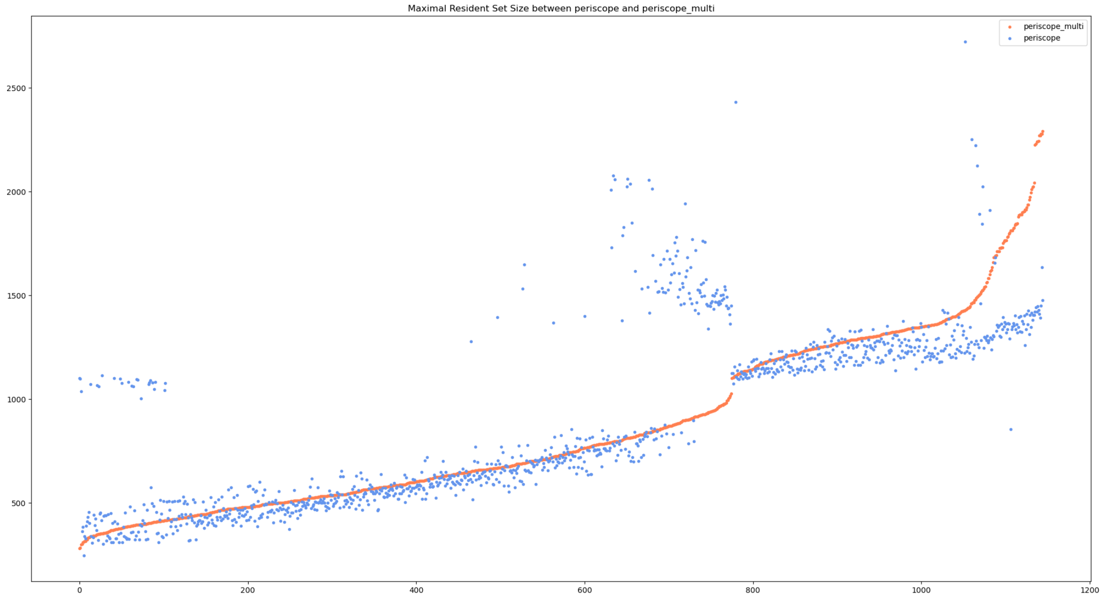

Figure S6. Maximal Resident Set Size time `periscope` and `periscope_multi` for each sample of the BIO-large dataset The blue point correspond to `periscope` result and the orange to `periscope_multi` . Each point represent the Maximum RSS for one of the sample of the Bio-large dataset. The samples are sorted in ascending order of size with `periscope_multi`

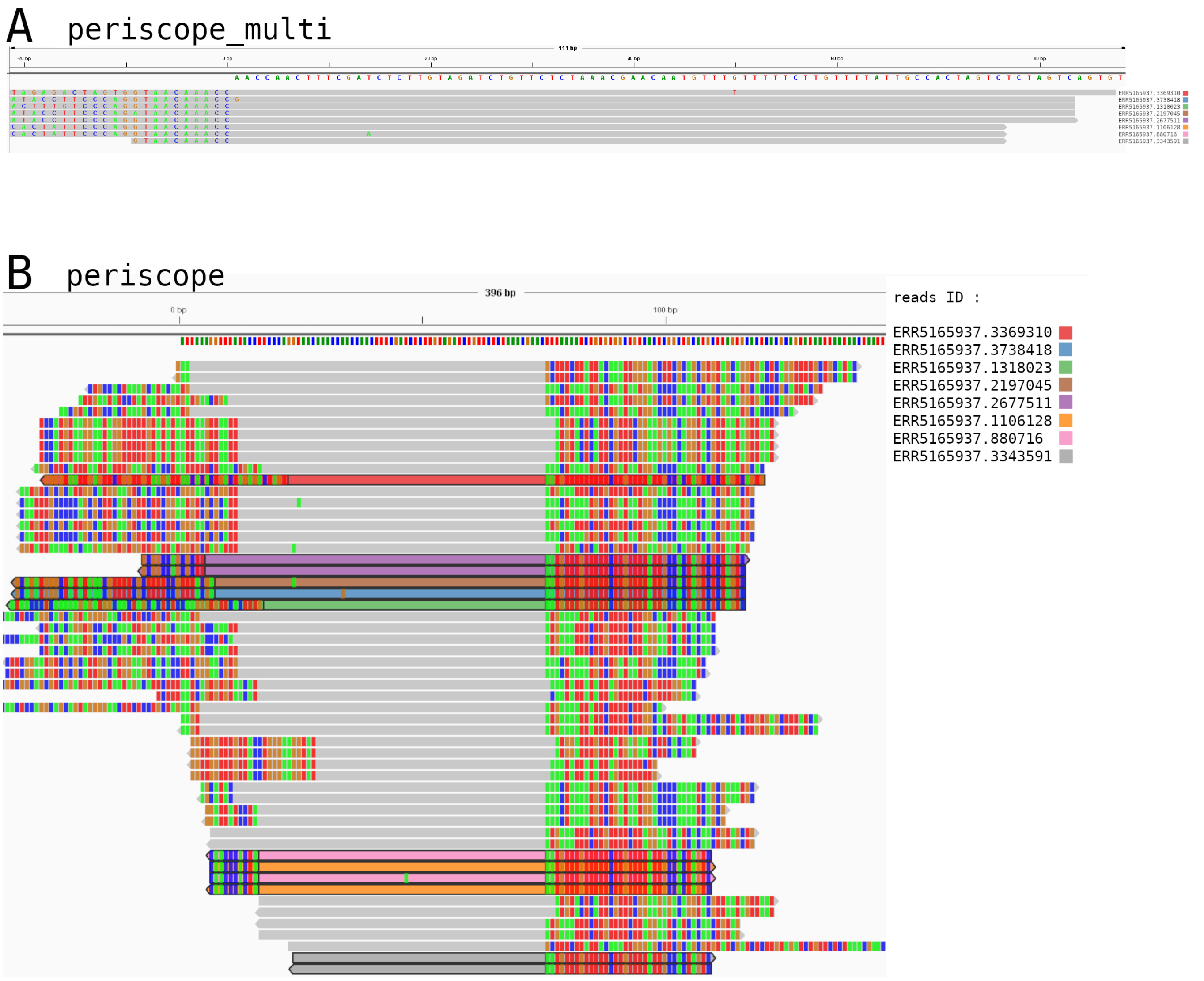

Figure S7. Picture of the different alignments obtained by `periscope` and `periscope_multi` for the BIO-ILLUMINA dataset. Figure shows alignment of eight reads found as canonical by `periscope_multi` (panel A) and classified as non canonical sgRNA by `periscope` . The same 8 reads are colored in the periscope alignments (panel B), and the corresponding read ID is displayed to the right.

REFERENCES

1. Lavezzari, D., Mori, A., Pomari, E., Deiana, M., Fadda, A., Bertoli, L., Sinigaglia, A., Riccetti, S., Barzon, L., Piubelli, C., et al. (2023) Comparative analysis of bioinformatics tools to characterize SARS-CoV-2 subgenomic RNAs. *Life Science Alliance,* **6**(12).
2. Di Gioacchino, A., Legendre, R., Rahou, Y., Najburg, V., Charneau, P., Greenbaum, B. D., Tangy, F., van Der Werf, S., Cocco, S., and Komarova, A. V. (2022) sgDI-tector: defective interfering viral genome bioinformatics for detection of coronavirus subgenomic RNAs. *RNA,* **28**(3), 277–289.
3. Sović, I., Šikić, M., Wilm, A., Fenlon, S. N., Chen, S., and Nagarajan, N. (2016) Fast and sensitive mapping of nanopore sequencing reads with GraphMap. *Nature communications,* **7**(1), 11307.
4. Li, H. (2013) Aligning sequence reads, clone sequences and assembly contigs with BWA-MEM. *arXiv preprint arXiv:1303.3997,*.
5. Wheeler, D. L., Barrett, T., Benson, D. A., Bryant, S. H., Canese, K., Chetvernin, V., Church, D. M., DiCuccio, M., Edgar, R., Federhen, S., et al. (2007) Database resources of the national center for biotechnology information. *Nucleic acids research,* **36**(suppl_1), D13–D21.
